# Techno-economic analysis of rooftop solar power plant implementation and policy on mosques: an Indonesian case study

**DOI:** 10.1038/s41598-022-08968-6

**Published:** 2022-03-21

**Authors:** Suparwoko, Fadhil Ahmad Qamar

**Affiliations:** 1 Department of Architecture, Islamic University of Indonesia, Yogyakarta, Indonesia; 2 Smart Energy Indonesia, Yogyakarta, Indonesia

**Keywords:** Environmental social sciences, Energy science and technology, Climate change

## Abstract

Indonesia is pushing the implementation of renewable energy to meet its climate action target. Solar energy is abundant, and its utilization is prioritized, including rooftop solar power plant (RSPP). This research presents a techno-economic analysis of an RSPP installed in a mosque in Ngombol subdistrict, Purworejo district, Central Java, Indonesia. This article also introduces and explains the regulation of RSPP and electricity tariffs in Indonesia, which define the economics of RSPP. This study employs an operational and financial model to analyze RSPP in five scenarios. The RSPP design objective is to reduce the annual energy usage of the mosque and yield the highest Net Present Value (NPV). According to the result, RSPP at all configurations based on the type and number of panels yield negative NPVs at the current electricity tariff, costs of components, and regulations implemented concerning RSPP. Proposed policy adjustment modeled through different scenarios provide benefit to some extent, limited by other policies. Hence, a combination of different policy adjustments may be required to achieve the most optimal condition for RSPP implementation on the mosque rooftop. This study could help policymakers to understand the possible directions of policy design for faster PV implementation.

## Introduction

In terms of the potential for renewables, Indonesia has a total capacity of 417.6 GW from different sources such as tidal, geothermal, bio, wind, hydro, and solar energy^1^. 207.8 GW or about half of this capacity is from solar energy thanks to its geographical condition crossed by the equator^2^. This capacity potential is relatively more significant than the total electricity generation capacity in June 2020, only at 71 GW^3^. By the first September 2021, solar energy implementation in Indonesia only accounts for 0.08% of its potential, with a capacity of 150 MWp^4^. This utilization rate is relatively low as Indonesia’s energy sector is still dominated by fossil fuels. In 2020, fossil fuel supported 88.8% of the primary energy supply and 85.3% of the electricity generation capacity^3,5^. This condition results in high Green House Gas (GHG) emissions from the energy sector, comprising around 30–40% of the total GHG emission in Indonesia^6^. Renewable energy has been known as a low-carbon alternative energy source to replace fossil fuels. Tawalbeh et al. estimated the carbon footprint from solar power to be in the range of 14–73 g CO2-eq/kWh, which is only 2 to 10% of the carbon emitted in oil-fueled electricity generation for generating one kWh^7^. Therefore as a part of its climate action, Indonesia targets to increase the share of renewable energy in its primary energy supply mix to 23% by 2025^1^.

To achieve this target, the Government of Indonesia (GOI) planned to increase the solar power plant (SPP) capacity to 3.6 GWp by 2025, comprising large-scale SPP, rooftop SPP (RSPP), and floating SPP^8^. The Institute for Essential Services Reform (IESR), a think-tank in energy and environment, suggested the possibilities of Indonesia achieving the energy transition goal by utilizing the distributed generation of solar power in the form of rooftop and off-grid SPP. IESR further estimated that a minimum potential of 2 GWp RSPP exists in Jakarta and Surabaya’s residential market^9^. This study implies a more significant potential when considering other cities and types of buildings. A growing interest in studies on RSPP design can also be seen in Indonesia, which analyzes RSPP on residential^10,11^, office^12,13^, educational^14–16^, and industrial buildings^13^. This paper will focus on SPP on mosque rooftops due to its significance in the context of Indonesia, while this type of building has not received the same research attention as others.

Indonesia is one of the countries with the largest population of Muslims in the world. For that reason, the Ministry of Religious Affairs reported the existence of more than 600,000 mosques and prayer halls of different sizes to support their activities^17^. Muslims gather daily at mosques for congregations at five designated times (before dawn, midday, afternoon, sunset, and evening), with a higher volume of visits every Friday midday. The sheer number of mosques suggests a large cumulative rooftop area, while the recurrent use of mosques may result in significant electricity use, which justifies the potential for RSPP implementation. In 2017, the Vice President of Indonesia and the Indonesian Council of Clerics, Majelis Ulama Indonesia (MUI) launched the eco-mosque initiative that aims to increase the environmental awareness of communities through renewable energy use, energy efficiency, waste management, and water preservation in mosques^18^. The relatively large area of the mosque’s roof is suitable for RSPP installation. The RSPP can provide the mosque with clean electricity with relatively low operation and maintenance expenditure (OPEX), resulting in electricity bills and GHG emission reduction from the financial and environmental perspectives. Higher electricity tariffs and the technology’s decreasing capital expenditure (CAPEX) result in RSPP’s more attractive business case^19^. According to Ghazali et al., the use of clean energy in such buildings is also ethically and morally relevant since preserving nature and the environment is one of the teachings of Islam, as indirectly suggested by the religion’s holy book^20^.

In the broader context, the adoption of solar PV suits well for rural electrification through micro-grid. Due to its decentralized nature, the micro-grid is an economical option to enable electrification in remote areas than extending the centralized grid^21,22^. The micro-grid with the decentralized generation, such as SPP, yields several advantages such as energy loss reduction, a more reliable supply, and carbon emission reduction due to the higher share of renewable energy adoption^23,24^. Mosques ideally serve as places of worship and as community building centers for their surroundings^25^. Especially for rural areas, this purpose can be further enhanced by coupling mosques with decentralized generation and connecting them through micro-grid^26,27^.

There has been a significant number of researches on the implementation of rooftop solar PV for mosques, as will be briefly discussed in this paper. Rashid et al. showed RSPP’s ability to reduce the annual electricity bill by 47% in Malaysia^28^. The payback period for the system’s CAPEX was reached in 13 years. A similar study was also conducted in Kuwait by Almutairi to identify the financial feasibility of the RSPP at 1,400 mosques in 2018, and the exact payback period was recorded^29^. The PV systems were designed to supply the connected load and shave the peak load during the day. It is, however, essential to note that both studies did not consider the present value of future cash flow, which means that the benefit of the system is subject to overestimation with the actual payback period achieved at a later date.

In 2019, Elshurafa et al. conducted a more comprehensive pilot study of the mosque RSPP in Saudi Arabia^19^. The RSPP was connected to the grid for different scenarios, including no supporting policy and a net-metering mechanism enabling the compensation for the exported surplus generation to the grid. The results showed that the analyzed system was financially attractive for both scenarios. The study finds that the analyzed system is economically attractive for both scenarios. The implementation of net-metering reduced the cost incurred for fulfilling electricity needs by 22% lower than the RSPP with no support policy scenario.

The GOI has implemented regulation on the RSPP through the Ministerial Regulation of the Minister of Energy and Mineral Resources (MEMR) No. 49/2018 to support the implementation of this technology^30^. Among other points, this regulation includes a net-metering scheme for RSPP in Indonesia, enabling consumers with RSPP to export their excess produced energy to the grid owned by the State Electricity Company, Perusahaan Listrik Negara (PLN). The exported electricity will offset the imported electricity from PLN and be calculated each month. The amount of electricity a consumer can offset every month is 65% of the exported electricity. PLN argues that this arrangement is made to account for transmission and distribution expenses^31^. In the case of higher export, the export balance can be accumulated for three months before the balance is reset to zero. This regulation also set the maximum capacity of RSPP, which is 100% of the electricity capacity stated in the contract. However, reports suggest that despite the Government’s intention to accelerate the implementation of RSPP, this regulation fails to enable the financial benefits of installing RSPP. Based on the simulation done by the Institute for Energy Economics and Financial Analysis (IEEFA), consumers will face difficulties in estimating their size of SPP to maximize savings, which is also sensitive to the consumers’ daytime load profile^31^. Their result shows that the policy favors consumers with high daytime load profiles and minimal export to the grid, contrary to its aim, which encourages RSPP through the ability to export to the grid. This load profile is uncommon as the majority of the consumers, especially in the residential sector, have a higher load profile in the evening.

Setyawati examines the perception towards the current regulation on RSPP in Indonesia through interviews and online surveys^32^. The survey results indicate that both consumer and institutional barriers constrain the RSPP implementation under the current policy. 71% of 987 PLN customers who participated in the surveys are interested in installing RSPP but prefer to wait for other options, which implies the less attractiveness of the current policy. The consumer is concerned about the high capital cost, long-term return on investment, and lack of information. Through the interviews with the Government, private sector, and energy experts, Setyawati identified that the low export rate for electricity is a substantial hurdle to attracting prospective users.

The initial investment in CAPEX and electricity bill savings, which is a function of the electricity tariff, define the return on investment of SPP in general, including RSPP. In Indonesia, the MEMR determines the electricity tariff provided by the PLN through the Ministerial Regulation, which is kept updated from time to time^33–36^. There are 37 electricity tariffs grouped by the consumer sector (Residential, Social, Industrial, Business, and Governmental) and the size of electricity capacity, expressed in VA^37^. Among the 37 tariff groups, 25 of them are subsidized, including the electricity tariff for all consumers in social. Compared to the basic production costs of electricity generation, Biaya Pokok Produksi (BPP), determined through the MEMR Ministerial Decree, the electricity tariff for the social sector is around 35% lower^38^. Hence, the subsidized electricity tariff for social sector consumers may hamper the ability of RSPP on the mosque in Indonesia to recover its initial investment due to the lower bill savings. The regulation on electricity tariff also administers a minimum monthly usage of 40 h which prevents consumers from entirely avoiding electricity bills despite the sufficient RSPP production to offset the imports. Referring to the analysis of IEEFA, consumers in the social sector will find it even more challenging to determine the size of RSPP to maximize electricity bill savings.

There are various methods for sizing SPP. Several SPP design and analysis studies adopt commercially available software, such as Homer^12,19,39^, PVsyst^10,11,16,29^, SOLARGIS pvPlanner^10,14^, RETSreen^10^, and System Advisory Model (SAM)^13^. Šimić et al. proposed a method for sizing an economically optimal SPP based on Croatian policy^40^. The price of the electricity produced by the SPP depends on the ratio of export and import. The policy imposes lower electricity prices generated by the SPP and reduced income at a higher export ratio. Thus, oversizing the SPP has a detrimental effect on the financial feasibility of the SPP.

This study aimed to conduct a similar analysis with those previously mentioned in related research, which involves exploring the design and feasibility of a mosque RSPP based on a location and the regulation in effect on RSPP in Indonesia as the case study. As mentioned above, the current policies revolving around the implementation of RSPP in Indonesia are somewhat unsupportive. Thus, four different policy scenarios are proposed in the simulations to realize RSPP implementation’s financial benefit better. To that end, we present a detailed techno-economic analysis of mosque RSPP in Indonesia for the first time, and the findings are expected to serve two purposes. The first is to define the implementation of RSPP on a mosque in Indonesia. The second is to provide insights to policymakers on the effectiveness of the current supporting policy for implementing the system for a specific end-user.

This paper is structured into four chapters. Chapter 1 covers the introduction and literature reviews of related studies. In chapter 2, the methods and the assumptions adopted in this paper are elaborated. Chapter 3 presents the result of the design. Chapter 4 presents the discussion and the conclusion of this paper.

## Methods and assumptions

This chapter describes the assumptions based on the case study, followed by the methods employed, consisting of three main activities: data acquisition, RSPP design, and design result analysis. The analyses in each activity are done using codes written in MATLAB.

### Case study

**Figure 1:**
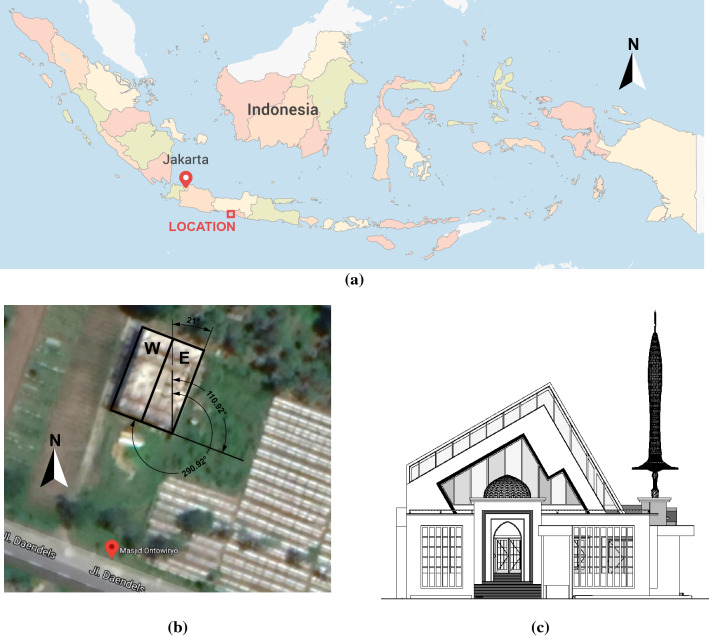
(**a**) Indoesia Map showing the location of the Ontowiryo Mosque, modoified from FreeVectorFlags.com with CorelDRAW 2018^41,42^, (**b**) Google maps image showing the mosque that is facing south-west. The mosque roof has two sides, west (W) and east (E). Image is modified with CorelDRAW 2018^42^, (**c**) The South Façade adapted from the mosque’s Detailed Engineering Design^43^.

The Ontowiryo Mosque is located at 109.96° Longitude, −7.85° Latitude, in Wonosari village, Ngombol subdistrict, at the south side of Purworejo district, Central Java. The configuration of the roof, azimuth, tilt, and area, is presented in Fig. 1 and Table 1. The mosque is connected to the grid with a maximum electricity capacity of 5,500 VA. As a community center, installing RSPP on the mosque further enhances its purpose, such as providing street lighting to its surroundings, given the lack of such service in the neighborhood of the case study. This purpose is of interest for further studies as the RSPP analyzed in this paper is limited to supplying the energy use in the mosque, described in the following section.

**Table 1:** Ontowiryo Mosque roof configuration.

	unit	West	East
Roof azimuth	(°, North = 0°)	291	111
Roof tilt	(m2)	30	60
Roof area	(°)	145.6	108.4

### Data acquisition

The data acquisition process prepares the required data for the RSPP design process, including site condition data and hourly electricity load profile data. The site condition data are obtained from Solcast, which provides a comprehensive set of hourly site condition data^44^. This data includes air temperature, cloud opacity, Diffuse Horizontal Irradiance (DHI), Direct Normal Irradiance (DNI), Global Horizontal Irradiance (GHI), sun azimuth angle, sun altitude angle, wind speed, relative humidity, and precipitable water. Site condition data are available for the chosen location for 12 years (2008–2019) or 4,383 days, containing 105,192 hourly data.

**Figure 2:**
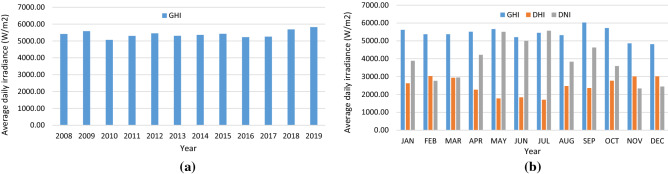
(**a**) average daily GHI at each year between 2008–2019. (**b**) Average daily irradiance (DHI, DNI, and GHI) for each month in 2008.

Figure 2a shows the average daily GHI across 12 years of data with a relatively constant value with an average of 5.4 kW/m^2^ per day. The slight fluctuation is due to the seasonal difference between years, where one year may have a longer dry/wet season and vice versa. The dry season in Indonesia spans in the middle of the year, while the rest is the wet season. Figure 2b presents the variation of irradiances throughout the year. GHI is relatively constant, while higher DHI values are observed at the end and beginning of the year. This condition is due to the wet season with more frequent rain and cloudy skies. Due to the relatively constant irradiance value across years, the site condition data used in the design and simulation can be represented by the data from one of the available years, with its average daily GHI being closest to the average. Thus, 2008, with an average daily GHI at 5.41 W/m^2^, is used for the design and simulation. Since 2008 is a leap year, only data from 365 days is used to simulate a year of operation.

The hourly load profile data is ideally generated from historical data by taking a sample of electricity consumption in one day. This method is unavailable for the considered case study as the mosque is currently under construction. Therefore, the load profile of the mosque is estimated by calculating the lighting and electronic equipment that is possibly used for specific activities and at certain times, presented in Tables 2 and 3. The list of lighting used in each room is obtained from the mosque’s Detailed Engineering Design. Electrical appliances used for the mosque operation include a sound system, desktop, router, refrigerator, vacuum cleaner, and water pump.

**Table 2:** Lightings number, power, location, and operational period.

Room	Lighting			Operational period
	points	Power (W)	Total (W)	
Mosque				
Main hall	7	72	504	Congregation time and 17.00—21.00 (8 h/day)
	2	18	36	
Porch	11	18	198	17.00–08.00 (15 h/day)
Office	2	18	36	08.00–18.00 (10 h/day)
Bedroom	2	18	36	17.00–08.00 (15 h/day)
Kitchen	3	18	54	Meal time (6 h/day)
Toilet (M)	4	18	72	Congregation time and 17.00—21.00 (8 h/day)
	1	23	23	
Toilet (F)	5	18	90	
Ablution (M)	1	18	18	Congregation time (5 h/day)
	1	23	23	
Ablution (F)	1	18	18	
	1	23	23

**Table 3:** Electrical appliances power and operational period.

Electrical devices	Brand	Power (W)	Operational period
Vacuum cleaner	Electrolux Z931	1,600	1 h/day
Water pump	Shimizu ZPS20-12-180	500	1 h/day
Desktop	(-)	150	08.00—18.00 (10 h/day)
Router/modem	TL-MR3420	12	24 h/day
Refrigerator	Aqua AQR-D190-DS	80	24 h/day
Speaker column	MAP-403SC “MAXX AUDIO PRO”	80	Congregation time (2.5 h/day)
Speaker horn	TOA – ZH 5025 BM	50	
Power amplifier	TOA Mixer Amplifier ZA-2120	120	
Sound Mixer	MG10XUF	23	

**Figure 3:**
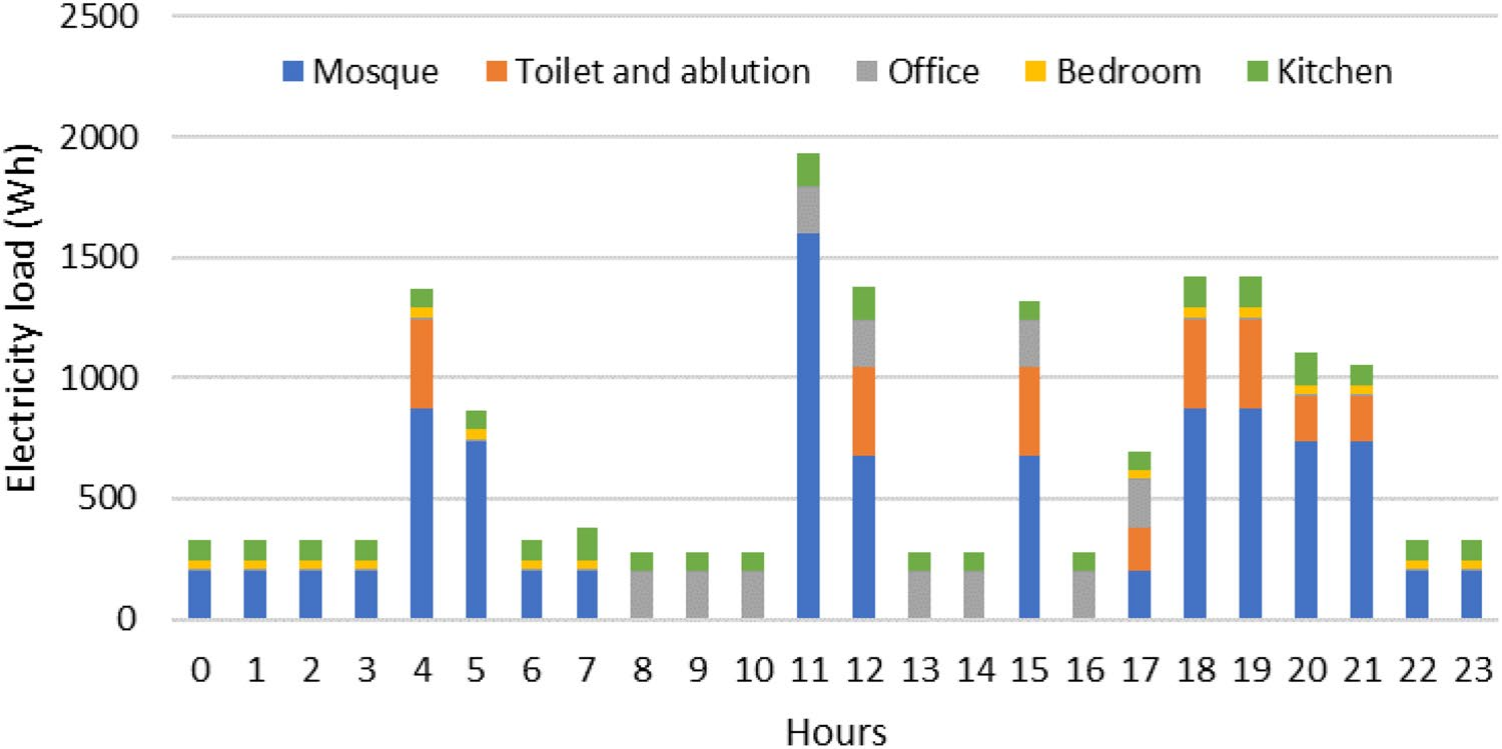
The hourly load demand profile of Ontowiryo Mosque.

The final load profile of the mosque is presented in Fig. 3. The electricity use of the mosque is 16.89 kWh/day and 6,166.49 kWh/year. To conclude, this research work with 8,760 hourly data simulates a full year operation of RSPP.

### RSPP design and analysis

The solar irradiance received by the rooftop area arrives in three forms: direct irradiance (${G}_{M}^{dir}$), diffused irradiance (${G}_{M}^{dif}$), and irradiance reflected by the ground (${G}_{M}^{ground}$)^45^. The solar irradiance data obtained in the previous activity (DNI, DHI, and GHI) are used to calculate each value of these forms. Equation (1) defines the direct irradiance where $\gamma$ is the angle of incidence (AOI), the angle between the PV module surface normal, and the incident direction of the sunlight. Given the sun’s position that constantly changes, AOI is a function of panel azimuth angle ${A}_{M}$, panel height angle ${a}_{M}$, sun azimuth angle ${A}_{S}$ and the sun elevation angle ${a}_{S}$. Equation (2) define the $\mathrm{cos}\gamma$.

1 $${G}_{M}^{dir}=DNI \cdot \mathit{cos}\gamma$$

2 $$\mathit{cos}\gamma =\mathit{cos}{a}_{M}\mathit{cos}{a}_{S}\mathit{cos}\left({A}_{M}-{A}_{S}\right)+\mathit{sin}{a}_{M}\mathit{sin}{a}_{S}$$

Equation (3) defines the diffused irradiance, a function of the Sky View Factor (SVF). SVF is the fraction of the sky from which the module can receive diffused irradiance expressed in Eq. (4). Equation (5) defines the irradiance reflected by the ground where $$\alpha$$ is the albedo of the ground which determine the reflectivity coefficient of the ground. The total irradiance received by the module ($${G}_{M})$$ is obtained by summing the three components of irradiance to determine the irradiance received by the rooftop and, hence, the estimated yield of solar energy on the mosque roof.

3 $${G}_{M}^{dif}=DHI \cdot SVF$$

4 $$SVF=\frac{1}{2}\left(1+\mathit{cos}{\theta }_{M}\right)$$

5 $${G}_{M}^{ground}=GHI \cdot \alpha \cdot (1-SVF)$$

6 $${G}_{M}={G}_{M}^{dir}+{G}_{M}^{dif}+{G}_{M}^{ground}$$

**Figure 4:**
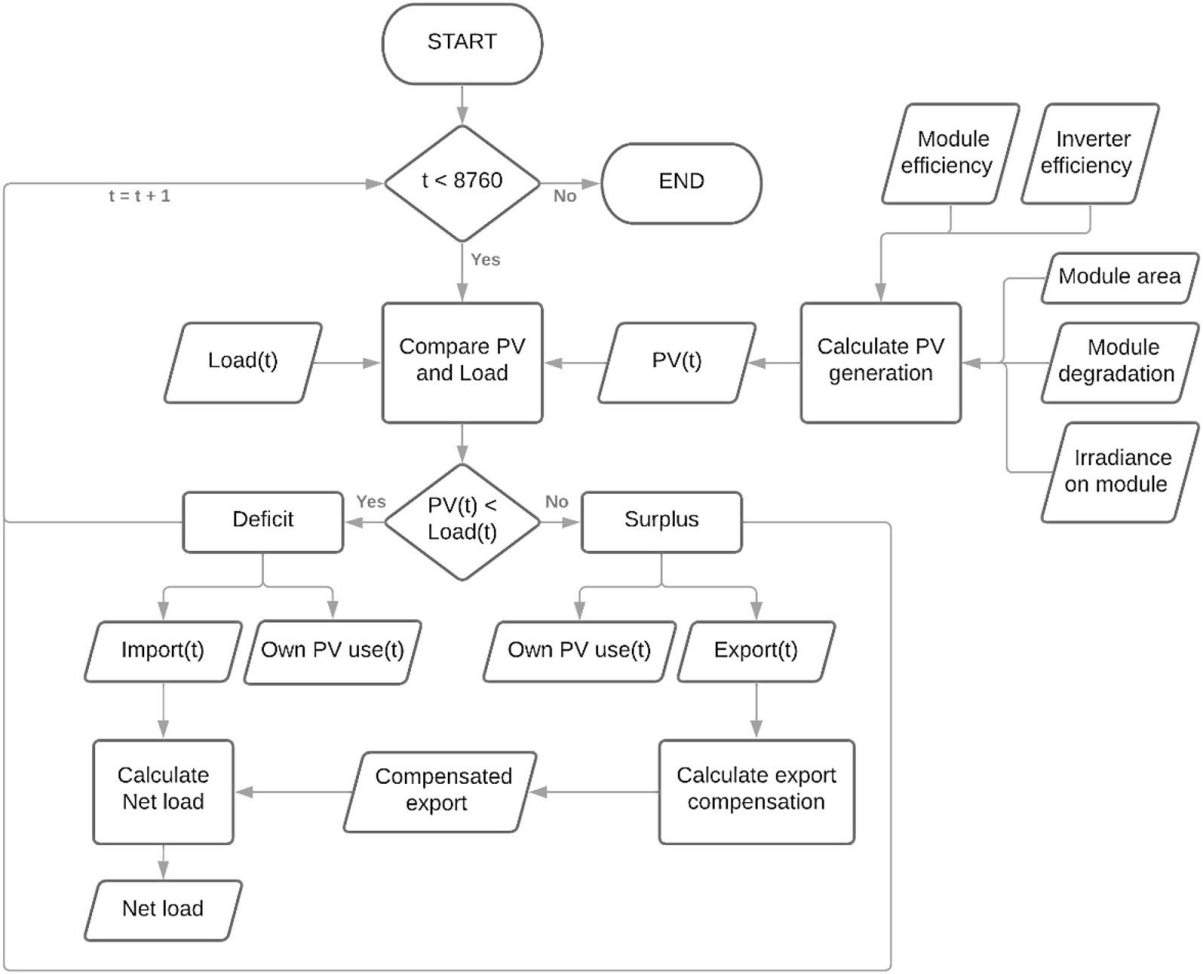
RSPP operational model.

This paper studied a grid-connected RSPP modeled in Fig. 4. The system is modeled in the hourly time step for one year, containing 8760 data steps, and repeated for 25 years (2021–2045) of the RSPP assumed lifetime^46^. RSPP generation in one hour is calculated as a product of the area of the module, module number ($${n}_{M}$$), irradiance received by the module ($${G}_{M})$$, inverter efficiency ($${\eta }_{inv}$$), module degradation rate ($${\eta }_{deg}$$), and module efficiency ($${\eta }_{M}$$), presented in Eq. (7). Module efficiency is a function of the module’s irradiance level and temperature ($${T}_{M})$$ as presented in Eq. (8) until 12^45^.

7 $${E}_{RSPP}(t)={\eta }_{M}\left({T}_{M},{G}_{M}\right) \cdot {\eta }_{inv} \cdot {\eta }_{deg} \cdot {area}_{M} \cdot {n}_{M} \cdot {G}_{M}$$

8 $${\eta }_{M}\left({T}_{M},{G}_{M}\right)={\eta }_{M}\left(25^\circ{\rm C} , {G}_{M}\right) \cdot \left[1+\kappa \left({T}_{M}-{T}_{STC}\right)\right]$$

9 $${\eta }_{M}\left(25^\circ{\rm C} , {G}_{M}\right)=\frac{FF \cdot {V}_{oc}\left(25^\circ{\rm C} , {G}_{M}\right) \cdot {I}_{sc}\left(25^\circ{\rm C} , {G}_{M}\right)}{{G}_{M} \cdot {area}_{M}}$$

10 $${V}_{oc}\left(25^\circ{\rm C} , {G}_{M}\right)={V}_{oc}\left(STC\right)+\frac{n{k}_{B}{T}_{STC}}{q}\mathit{ln}\left(\frac{{G}_{M}}{{G}_{STC}}\right)$$

11 $${I}_{sc}\left(25^\circ{\rm C} , {G}_{M}\right)={I}_{sc}\left(STC\right)\left(\frac{{G}_{M}}{{G}_{STC}}\right)$$

12 $$FF=\frac{{I}_{mpp} \cdot {V}_{mpp}}{{I}_{sc} \cdot {V}_{oc}}$$

$$\eta \left(25^\circ{\rm C} , {G}_{M}\right)$$, $${V}_{oc}\left(25^\circ{\rm C} , {G}_{M}\right)$$, and $${I}_{sc}\left(25^\circ{\rm C} , {G}_{M}\right)$$ are the module efficiency, open-circuit voltage, and short circuit current as a function of irradiance level. Data obtained from the module’s datasheet are temperature coefficient ($$\kappa$$), open-circuit voltage (V_oc_), short circuit current (I_sc_), area of the module, and module’s degradation rate. The values for Boltzmann constant (k_b_), elementary charge (q), and quality factor (n) are obtained from^47^. FF is the Fill Factor, the ratio between power generated at maximum power point and the product of V_oc_ with I_sc_. G_STC_ and T_STC_ are the standard conditions for irradiance and temperature when the PV modules were tested, at 1000 Wm^−2^ and 25 °C. The module’s temperature ($${T}_{M})$$ is determined through the fluid-dynamic model which considers heat sources from sun irradiance, convective and radiative heat exchange from the front and rear side of the module. Equations (13–25) present the calculation for $${T}_{M}$$,

13 $${T}_{M}(t)=\frac{{\alpha }_{M}{G}_{M}+{h}_{c}{T}_{a}+{h}_{r,sky}{T}_{sky}+{h}_{r,gr}{T}_{gr}}{{h}_{c}+{h}_{r,sky}+{h}_{r,gr}}$$

14 $${h}_{c}={h}_{c}^{T}+{h}_{c}^{B}$$

15 $${h}_{c}^{T}=\sqrt[3]{{h}_{forced}^{3}+{h}_{free}^{3}}$$

16 $${h}_{forced}= {w}^{0.8}$$

17 $${h}_{free}= \frac{0.21 \cdot k{(Gr \cdot Pr)}^{0.32}}{{D}_{h}}$$

18 $$Gr=\frac{g\left({T}_{M}-{T}_{a}\right){D}_{h}^{3}}{{v}^{2}}$$

19 $${D}_{h}=\frac{2LW}{L+W}$$

20 $${h}_{c}^{B}=R \cdot {h}_{c}^{T}$$

21 $$R=\frac{{\alpha }_{M}G-{h}_{c}^{T}\left({T}_{INOCT}-{T}_{a}\right)-{\epsilon }_{top}\sigma \left({T}_{INOCT}^{4}-{T}_{sky}^{4}\right)}{{h}_{c}^{T}\left({T}_{INOCT}-{T}_{a}\right)+{\epsilon }_{top}\sigma \left({T}_{INOCT}^{4}-{T}_{sky}^{4}\right)}$$

22 $${\alpha }_{M}=\left(1-R\right)\left(1-{\eta }_{M}\left(STC\right)\right)$$

23 $${T}_{INOCT}= {T}_{NOCT}+18$$

24 $${h}_{r,gr}={\epsilon }_{back}\sigma \left({T}_{M}^{2}+{T}_{gr}^{2}\right)\left({T}_{M}+{T}_{gr}\right)$$

25 $${h}_{r,sky}={\epsilon }_{top}\sigma \left({T}_{M}^{2}+{T}_{sky}^{2}\right)\left({T}_{M}+{T}_{sky}\right)$$

where ${\alpha }_{M}$ is the absorptivity of the module, ${T}_{a}$ is the ambient temperature, ${h}_{c}$ is the convective heat transfer coefficient of the module, ${h}_{r,sky}$ and ${h}_{r,gr}$ are the radiative heat transfer to the sky and the ground, or roof in case of RSPP, and ${T}_{sky}$ and ${T}_{gr}$ are the sky and ground temperature. The process of finding the module’s temperature is an iterative process as the ${h}_{r,sky}$ and ${h}_{r,gr}$ are functions of ${T}_{M}$. $Gr$ is Grashof number, $Pr$ is Prandtl number which equals to 0.71 for air, $\sigma$ Stefan-Boltzmann constant, ${D}_{h}$ is the hydraulic diameter of a rectangle with width $W$ and length $L$, $k$ is the air thermal conductivity and $v$ is the air viscosity. ${T}_{INOCT}$ is the installed nominal operating cell temperature which for direct mount equals to Eq. (23). ${\epsilon }_{back}$ and ${\epsilon }_{top}$ are the emissivity of the back and front surface of the module, which equals to 0.89 and 0.84 respectively.

**Figure 5:**
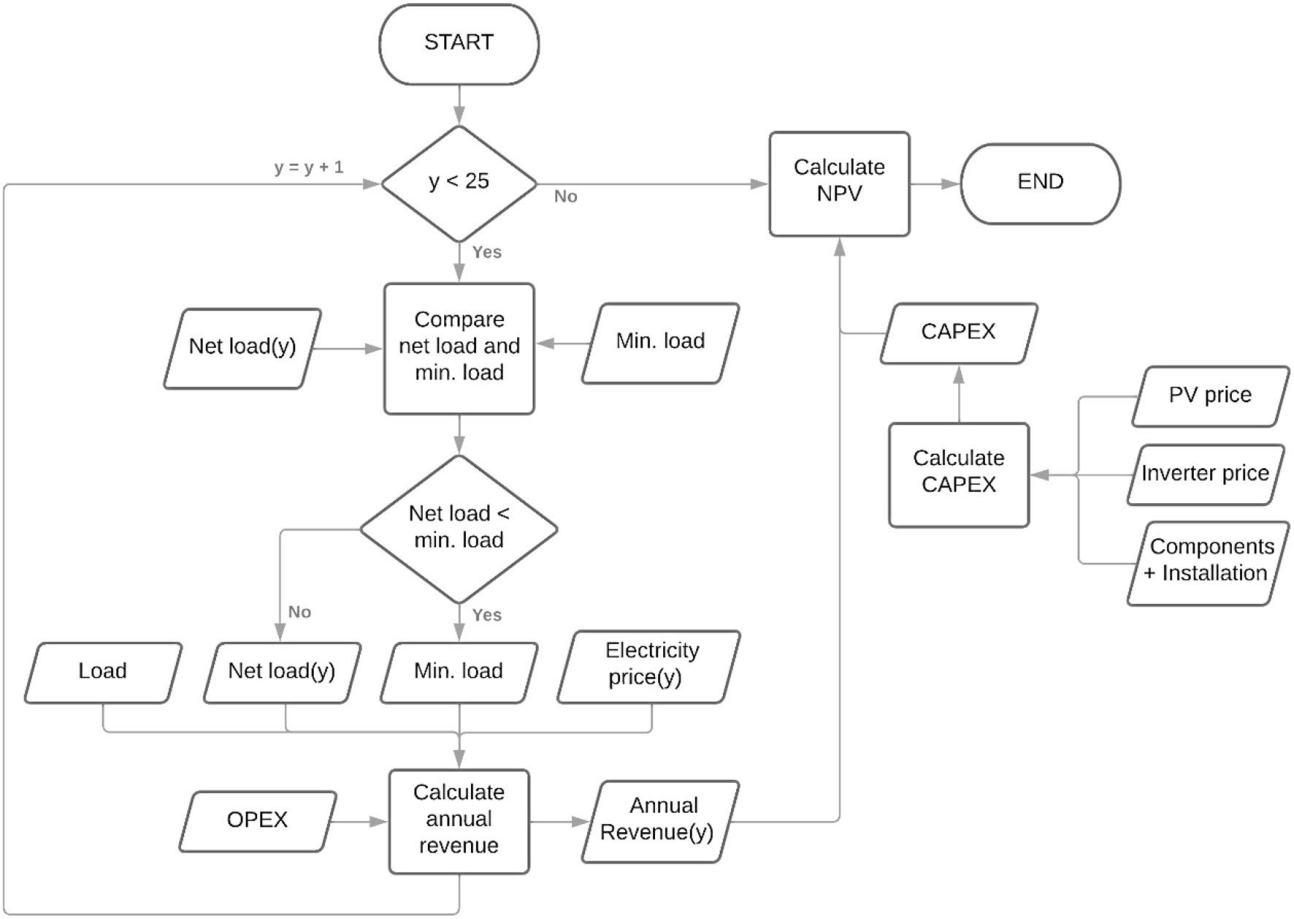
RSPP financial model.

The algorithm of the model compares the load and generated energy from RSPP every hour. When the load exceeds the RSPP generation, generated energy will be consumed while importing energy from the grid to supply the deficit. Vice versa, when the RSPP generation is higher than the load, the excess of the generated energy will be exported to the grid. Equations (26 and 27) present the import and export mechanism of the model. According to the regulation, the imports and exports are accumulated each month to determine the monthly electricity bill. PLN compensates 65% of the exported energy to offset the imported energy. The offset amount is limited that the reduced imported energy can not be lower than the monthly minimum load, equal to 40 h usage of the maximum electricity capacity, which results in 220 kWh monthly minimum load or 2640 kWh annual minimum load (${E}_{min load}$). If this limit is reached, the export excess will be deposited in the grid and offset up to the following three months’ imports before being reset to zero. Due to the limitation of the model, the offset balance reset mechanism is not modeled, and the model accumulated the imports and exports annually. Hence, the model may not account for the lost export deposits due to the reset mechanism. The net load, the reduced load due to the utilization of the RSPP, can be obtained by offsetting the imports with the compensated exports, expressed in Eq. (28). This value will be used in calculating the annual revenue of the RSPP, as further explained in the financial model in Fig. 5.

The RSPP gains its revenue from the annual electricity bill saving, calculated by subtracting the initial load with the final load after the utilization of RSPP multiplied with the electricity tariff ($${C}_{el}$$). In each year, the financial model compares the net load with the minimum load set by the regulation for 25 years of the RSPP assumed life span. The net load is a year function due to the module’s degradation rate, while the minimum load is a constant. If the net load is higher than the minimum load, the bill equals the net load. Vice versa, when the net load is below the minimum load, the bill equals the net load, and the excess is deposited in the grid. In practice, this excess can offset future imports when the RSPP generation is low. However, this model only uses one-year irradiance data and repeats them for the assumed life span of the RSPP. Hence, RSPP generation output is relatively constant, which RSPP with a relatively large capacity may result in unutilized deposited exports. The annual revenue of the RSPP ($$R(Y)$$), which accounts for the total cash inflow each year, is obtained from subtracting OPEX from the annual electricity bill saving. Darghouth et al. estimated that the CAPEX and OPEX for SPP in Indonesia is 1365.76 US$ and 24.38 US$/kW per year^48^. The annual revenues and the CAPEX of the RSPP are then used to calculate the system’s Net Present Value (NPV). Based on a market study in 2017, a typical proportion of RSPP CAPEX in Indonesia is consisting of 47% PV modules cost, 13% inverter cost and 40% other complementary components as well as installation cost^49^.The cost for PV modules and inverter in this paper are taken from a renewable energy technology distributor website since the estimate from Dargouth et al. lacks technical specification details needed for the simulation^50^. Just as other renewable energy technologies, RSPP is capital intensive. Therefore, the PV module with the lowest price/kW is employed to keep the CAPEX down and this study employs Suntech STP375S 375 W. Each module was modeled in the simulation at various RSPP capacities with the maximum electricity capacity as the upper limit or at 5.5 kWp. This study also employs 9 inverter types from Growatt of different sizes by matching the power output requirement of each simulation.The CAPEX includes PV modules ($${C}_{PV}$$), inverter ($${C}_{inv}$$) and other components and installation ($${C}_{other}$$) as presented in Eq. (31). Table 4 presents the price/kW of the PV module, inverter and other costs used in the calculation. The technical specifications of the PV modules and inverters can be found as Supplementary Table S1 and S2 online.

**Table 4:** CAPEX components price per capacity.

Capacity (kWp)	${C}_{PV}$(USD/kWp)	${C}_{inv}$(USD/kWp)	${C}_{other}$(USD/kWp)
< 1.05	350.77	336.76	= 40% × 1365.76 = 546.30
1.05 – 1.40		264.54	
1.40 – 2.10		186.11	
2.10 – 2.60		159.53	
2.60 – 2.80		143.59	
2.80 – 3.50		137.39	
3.50 – 4.20		159.56	
4.20 – 5.04		140.35	
5.04 – 5.88		129.80	

**Figure 6:**
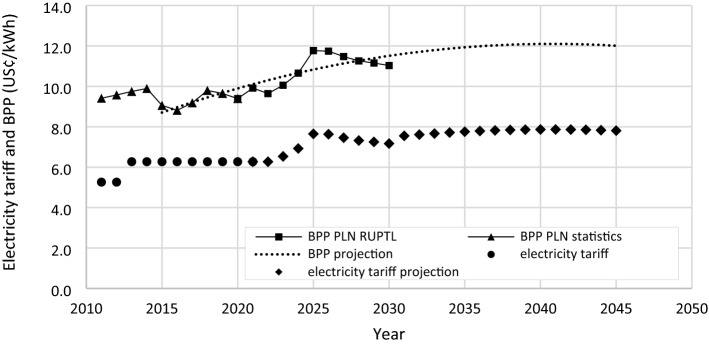
BPP and electricity tariff for case study.

The MEMR Ministerial Regulation regulates the electricity tariff in Indonesia. For social sector consumers with 5500 VA electricity capacity, the tariff has been 6.27 US¢/kWh for the last eight years. This tariff is subsidized as it only accounts for 65% of the BPP on average. The statistics report of PLN presents The BPP values from 2011–202,048,49, while the PLN’s Electricity Supply Business Plan (RUPTL) presents the projection of BPP values from 2021 to 2030^50^. The BPP values beyond 2030 are estimated using a second-order polynomial regression function generated from the known values of BPP from 2015–2030. The BPP drop in 2015 was due to the increase in power system efficiency^51^. The BPP spike in 2025 is due to the significant increase in renewable energy adoption to achieve the Paris Agreement in that particular year. It is estimated that the BPP will rise at a slower rate due to the coal PP phase-out starting in 2030 and the decreasing cost of renewable energy technologies^52^. Thus, the projected electricity tariff from 2022 onwards is set at 65% of the BPP presented in Fig. 6. The currency exchange rate used in this paper is 1 USD = 14,351 IDR and 1 GBP = 1.33 USD.

26 $${E}_{import}\left(t\right)=\left\{\begin{array}{l}{E}_{load}\left(t\right)-{E}_{RSPP}\left(t\right), {E}_{load}\left(t\right)>{E}_{RSPP}(t)\\ 0, {E}_{load}\left(t\right)\le {E}_{RSPP}(t)\end{array}\right.$$

27 $${E}_{export}\left(t\right)=\left\{\begin{array}{l}{E}_{RSPP}\left(t\right)-{E}_{load}\left(t\right), {E}_{RSPP}\left(t\right)>{E}_{load}(t)\\ 0, {E}_{RSPP}\left(t\right)\le {E}_{load}(t)\end{array}\right.$$

28 $${E}_{net load}(Y)=\sum_{t=1}^{8760}({E}_{import}\left(t\right)-0.65 \cdot {E}_{export}(t))$$

29 $$R(Y)=\left\{\begin{array}{c}{E}_{net load}\left(Y\right) \cdot {C}_{el}\left(Y\right)-OPEX, {E}_{net load}\left(Y\right)>{E}_{min load}\\ {E}_{min load}\left(Y\right) \cdot {C}_{el}\left(Y\right)-OPEX, {E}_{net load}\left(Y\right)\le {E}_{min load}\end{array}\right.$$

30 $$OPEX=24.38\left({I}_{mpp} \cdot {V}_{mpp} \cdot {n}_{M}\right)/1000$$

31 $$CAPEX={C}_{PV}+{C}_{inv}+{C}_{mount}+{C}_{work}$$

The financial model economic analysis employs the Net Present Value method, which estimates the project outcome economic value: positive or negative. The NPV integrates the initial investment and the expected incomes and costs that occur during the operation of the RSPP into a series of cash flows adapted to the time value of money and risk. Equation (32) presents the calculation for NPV for the 25 years of the RSPP operational life span^53^. The discount rate ($r$) is determined from the weighted average capital cost (WACC). IRENA assumes a real WACC of 7.5% in OECD countries and China and 10% elsewhere globally for all types of technology^54^. The IEA assumes a WACC of 8% in developed countries and 7% in developing countries^55^. Steffen estimated that the WACC for PV system development projects in and outside the OECD countries was 5.4% and 7.4%56. This paper employs a WACC level of 7% for the discount rate.

32 $$NPV= -CAPEX+\sum_{Y=0}^{25}\frac{R(Y)}{{\left(1+r\right)}^{Y}}$$

### RSPP design scenarios

As mentioned in the introduction, the current ecosystem may not be optimal for implementing RSPP on a mosque. Hence, several design scenarios are employed in the operational and financial model to explore the financial feasibility options.

- The business as usual (BAU) scenario models the RSPPs with two objectives: to have the highest NPV and to meet the load that is not covered with the minimum load limit.
- The second scenario, the carbon pricing (CP) scenario, models the RSPPs with additional income from the monetized carbon emission reduction. Climate Transparency estimated that approximately 761 g of carbon dioxide are emitted into the air when generating 1 kWh of electricity in Indonesia^57^. The Environmental Protection Agency (EPA) uses three sets of social cost estimates of carbon emissions (SCC) with different discount levels, as can be seen in Table 5 SCC^58^ is the discounted monetary value of the future climate change damages due to additional metric tons of carbon dioxide (CO2) emissions^59,60^. The money collected from the carbon pricing can also fund the energy transition and subsidize renewable energy implementation. This paper uses the SCC at 5% discount factor, closest to the discount factor used in the financial model.
- The third scenario is the elimination of the minimum load limit (MLL). The minimum load limit prevents the RSPP from entirely supplying the load. This scenario entails removing the minimum imports requirement. Such support policy was temporarily implemented in August 2020 through a MEMR Ministerial Decree to deal with the impact of COVID-19^61^.
- The fourth scenario is the rework of the Net-Metering Scheme (NMS). In this scenario, the NMS is reworked to compensate 100% of the exported energy. The GOI has planned this adjustment by revising Ministerial Regulation No. 49/2018 on RSPP in 2021^62^.
- The fifth scenario entails the enforcement of a non-subsidized electricity tariff (NST). The higher electricity tariff may drive customers to switch their means to meet their energy needs while enabling RSPP to gain more income. In this scenario, the BPP values will be used as the electricity tariff.

**Table 5:** SCC in US$/ton of carbon emission at different discount factors.

Discount Factor	Year					
	2020	2025	2030	2035	2040	2045
5.00%	12	14	16	18	21	23
3.00%	42	46	50	55	60	64
2.50%	62	68	73	78	84	89

**Table 6:** Irradiance potential on Ontowiryo mosque rooftop.

	unit	West	East
Roof azimuth	°, North = 0°	291	111
Roof tilt	°	30	60
Roof area	m^2^	145.6	108.4
Irradiance	kWh/m^2^ per year	1,808	1,302
	ESH	4.9	3.5
Total potential	MWh per year	263.24	141.7

## Result

This section presents the results on the irradiance potential on the mosque roof and the case studies in which the RSPP operational and financial model is applied to a mosque in several modeling scenarios. The RSPPs are modeled with capacities between 0.75 and 5.25 kWp, which employs 2–14 modules respectively. The CAPEX for these systems ranges between 1000.7 and 1233.8 US$/kWp.

### Roof irradiance potential

The site condition analysis shows that the solar irradiance potential in the case study location is at 1,971 kWh/year.m^2^ or equals to 5.4 daily Equivalent Sun Hours (ESH). This potential is achieved with an optimal solar panel tilt angle and azimuth configuration at 10° and 6°. The potential for each side of the roof is presented in Table 6. The west side roof has a higher irradiance potential due to its azimuth and tilt angle proximity to the optimal. Hence, RSPP designs in the following sections will use the west side roof parameters. The result also shows that the energy yield for this particular roof configuration 1374.7 kWh/kWp, Since mosque, including the case study, are typically built in a particular direction for religious activity purposes, this energy yield may represent the estimate for RSPP on mosque rooftop.

### BAU scenario

**Figure 7:**
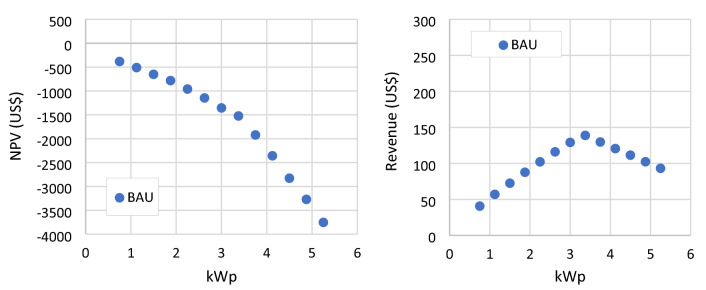
NPV and annual revenue of RSPP employing different numbers of PV modules in BAU scenario.

**Figure 8:**
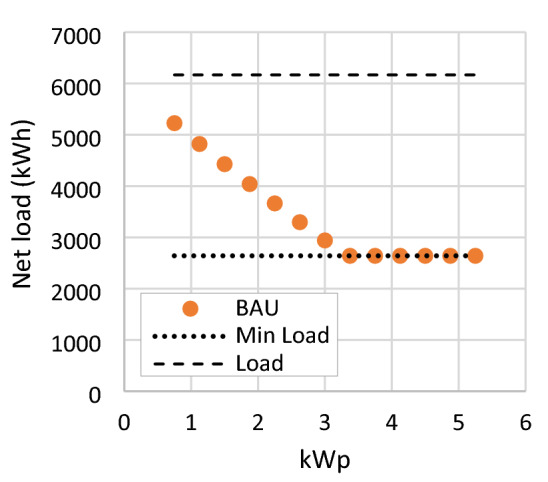
Net load of RSPP employing different numbers of PV modules in BAU scenario.

The result shows negative values for all PV modules numbers and types in the BAU scenario. RSPP with a capacity of 0.75 kWp reach the highestNPV of −382 US$, shown in Fig. 7. Annual revenue is maxed at 138.8 US$ with a 3.375 kWp RSPP. RSPP with this capacity will result a net load of 2600.68 kWh, which then capped by the minimum load limit of 2,640 kWh as presented in Fig. 8. At higher modules number, RSPPs suffer from annual revenue reduction due to the bill saving capped by the minimum load limit while the OPEX keeps increasing. This condition further decreases the NPV at a higher rate. A negative NPV denotes the inability of the annual bill saving from RSPP utilization to return the CAPEX.

### CP scenario

**Figure 9:**
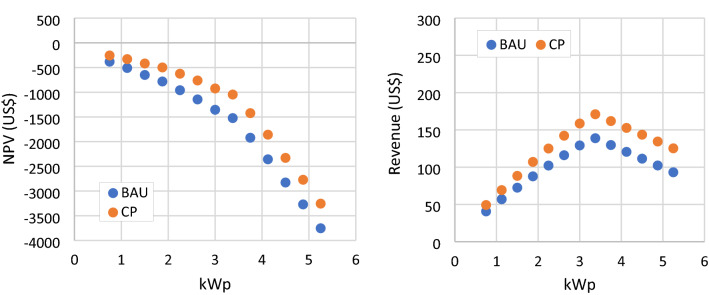
NPV and annual revenue of RSPP with different number of PV modules in BAU and CP scenario.

The enactment of carbon pricing in this scenario serves as an additional income when calculating the annual revenue of the RSPP. The increase of NPV values is visible in Fig. 9, but all PV modules numbers and types still have negative NPV values. In this scenario, RSPP with a capacity of 0.75 kWp yield the highest NPV of –257 US$, which can save cumulatively 17.9 tons of CO_2_e over its operational lifetime. The net load is not affected, and revenue reduction is also occurring at RSPP with higher capacities. The increasing gap between BAU and CP explain the increased revenue and NPV due to the carbon pricing policy. This condition implies that higher RSPP capacity will benefit more from carbon pricing scheme. However, when the minimum load limit is reached, higher capacity RSPP yield no additional amount of carbon emission reduction. Thus, that the gap between BAU and CP become parallel.

### MLL scenario

**Figure 10:**
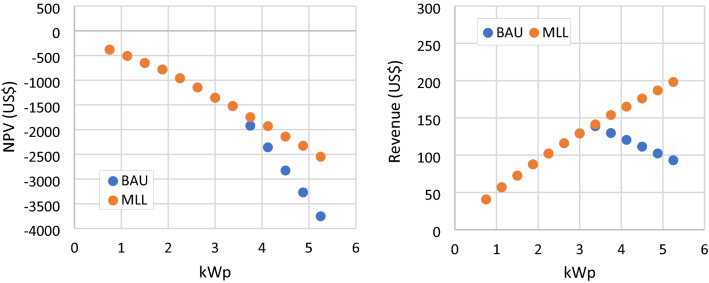
NPV and annual revenue of RSPP with different number of PV modules in BAU and MLL scenario.

**Figure 11:**
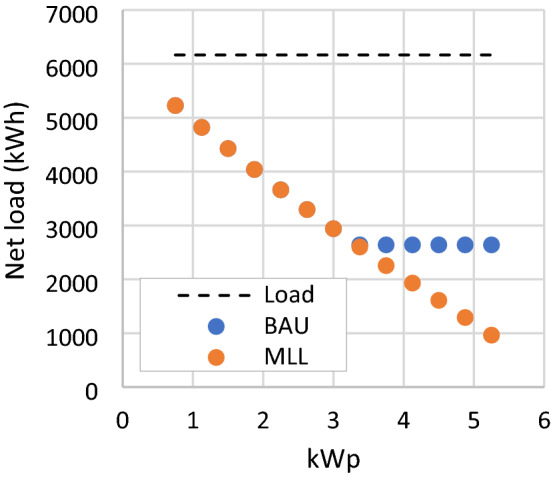
Net load of RSPP with different number of PV modules in MLL scenario.

The BAU scenario modeling found that the current regulation on the minimum load limit incurs a detrimental effect on the RSPPs’ financial attractiveness at higher capacities. Therefore, in this scenario, the limit is removed. This policy adjustment allows the owner of RSPP to meet their entire electricity need from the RSPP directly and from the compensation of exports. The revenue of RSPP in this scenario continues to increase with the increase of RSPP capacity, as shown in Fig. 10. However, the NPV for all PV modules configurations remains negative and RSPP with the highest NPV is the same as the BAU case. As presented in Fig. 11, RSPP with the highest capacity of 5.25 kWp supplies 84.4% of the mosque load with NPV of −2,485 US$. In the BAU scenario, this configuration would yield a much lower NPV at −3573 US$.

### NMS scenario

**Figure 12:**
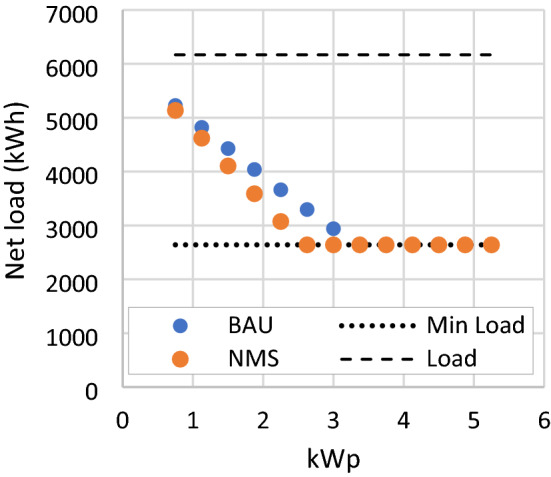
Net load of RSPP with different number of PV modules in BAU and NMS scenario.

**Figure 13:**
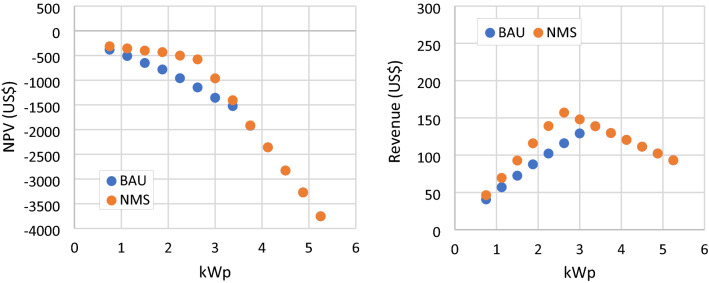
NPV and annual revenue of RSPP with different number of PV modules in BAU and NMS scenario.

In this analysis, the net metering scheme is reworked to compensate 100% of the exported energy from the PV system to the grid. Higher export compensation allows more load reduction from RSPPs with the same number of PV modules, shown in Fig. 12. This impact is more significant at a lower net load level, such as an RSPP with 11 PV modules under the NMS scenario can reduce the load as much as an RSPP with 14 modules under the BAU scenario. However, with the minimum load limit still in place, this scenario causes RSPP to exceed the limit at a lower RSPP capacity than in the BAU scenario. Thus, the additional load reduction is not applicable for RSPP when the load limit is exceeded, shown in Fig. 13. This scenario also results in higher annual revenue and NPV. RSPP with the highest NPV is the same as the BAU case with a slight increase of NPV, which is at −313 US$.

### NST scenario

**Figure 14:**
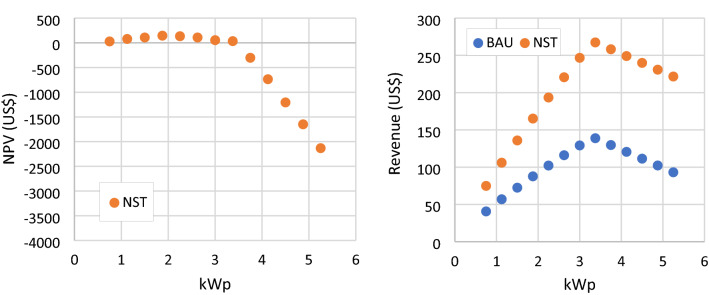
NPV and annual revenue of RSPP with different number of PV modules in BAU and NST scenario.

In this analysis, the electricity tariff is non-subsidized and taken from the BPP. This scenario only affects the revenue of different RSPP capacities, while the net load remains the same as BAU. In this scenario the revenue and NPV is increased, as presented in Fig. 14. Due to the higher electricity tariff, the utilization of RSPP results in higher bill saving. RSPP capacities lower than or equal to 3.375 kWp also achieve positive NPVs. RSPP with a capacity of 1.875 kWp yield the highest NPV of 144.44 US$.

**Figure 15:**
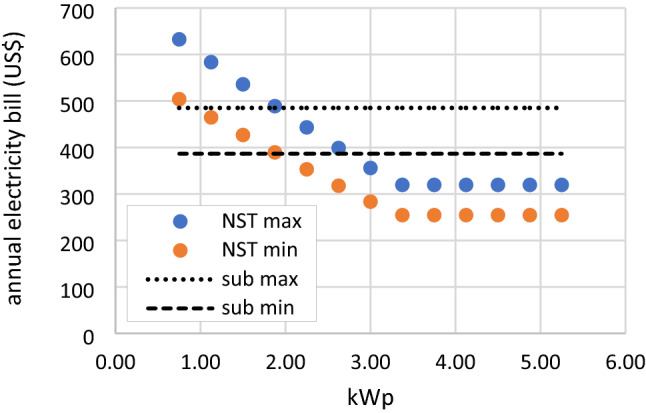
annual electricity bill for NST scenario and subsidized tariff scheme with no RSPP.

At some RSPP capacities, this scenario may also reduce the annual electricity bill, lower than the subsidized tariff scheme. Over the RSPP operational life span, the subsidized electricity tariff ranges between 6.27 and 7.87 US¢/kWh. Without any RSPP utilization, annual electricity bill of the mosque during this period ranges between 386.55 and 485.11 US$. Meanwhile the unsubsidized tariff ranges between 9.64 and 12.10 US¢/kWh. Hence, the annual electricity bill for different RSPP capacities under the NST scenario can be seen in Fig. 15. Despite the increased electricity tariff, the annual electricity bill of mosque with 3 kWp and 3.375 kWp RSPP are 355.86 and 319.52 US$, lower than those of in the subsidized tariff scheme. Higher capacities RSPP also result in lower annual bill but these systems have negative NPVs.

**Figure 16:**
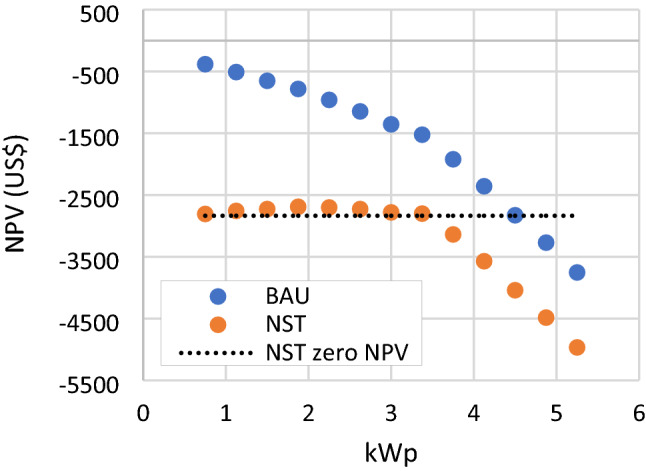
NPV of NST scenario compared to BAU scenario.

It is important to note that the NPV of the RSPPs in this scenario can not be directly compared with the NPV of RSPPs in BAU scenario presented in Fig. 7. Zero NPV may denote that the RSPP is able to return the investment. It also implies that investing in such RSPP would result in the same cost as meeting the load fully by importing electricity from the grid. Therefore, due to the tariff difference, the mosque will spend more money in NST scenario than in BAU in meeting the load. When incorporated, the present value of additional bill for 25 years of usage due to the increased electricity tariff is 2,834.23 US$. Thus, when compared to the current subsidized tariff condition, overall cost on meeting the load is higher in NST scenario. presents that RSPP can help reduce this cost slightly, as can be seen from the slight NPV increase for system with capacity lower and equal to 3.375 kWp. The highest NPV for the NST scenario in this analysis is −2,689.8 US$, which is reached with a 1.875 kWp RSPP as shown in Fig. 16.

## Discussion and conclusion

In this paper, we have assessed the design of RSPP on a mosque and its economic implications. Despite the carbon reduction benefit, at the current electricity tariff and capital costs of the components, and the implemented regulations around the adoption of RSPP, it is financially unfeasible to install a rooftop PV system on a mosque indicated by the negative NPV. The highest NPV of −382 US$ is achieved with a 0.75 kWp RSPP. The subsidized tariff of electricity caused lower annual revenue for RSPP to recover its CAPEX. The partial export compensation forces users to increase the RSPP capacity to get the same energy from an RSPP in a full export scenario. However, the minimum load limit regulation prevents users from installing high-capacity RSPP due to the capped revenue and the increasing OPEX that is a function of RSPP capacity. The results of this study reflect well with the study by Darghouth et al. that the low electricity tariff and minimum load limit hampers the financial attractiveness of RSPP in Indonesia. This study also broadens scope of the previous study by including analysis on customers in social sectors as well as different scenarios. The Indonesia’s less supportive regulatory structure on RSPP may result in the differing conclusions to existing studies on SPP integration on mosques in other countries where positive results were documented.

The result of this paper should serve policymakers as a reflection of the prevailing regulation on the adoption of PV in the power sector. This paper provides an insight that the plan to increase the share of renewable sources to 23% in the power generation by 2025 can be hampered by the prevailing regulation. Several policy adjustments were proposed and modeled to explore their implications on the financial attractiveness of RSPP:

1. Carbon pricing, export compensation rework and non-subsidized tariffs increase revenue and NPV as a function of the RSPP capacity until the minimum load limit is reached. The adjustment on minimum load limit should be prioritized to maximize the implementation of other supporting regulation.
2. The result shows that in the scope of the project, the enactment of non-subsidized tariff can increase the financial feasibility of RSPP indicated by the positive NPV and low annual electricity bill. However, the significant increase of electricity tariff shifts the present value down. Since the main hurdle of RSPP is the CAPEX, it is suggested to relocate the subsidy from electricity tariff to fund the deployment of RSPP. For the case study analyzed, the discounted amount of subsidy given by the government for 25 years of usage is around 2,834.23 US$. The same amount of money can fund a 2.8 kWp RSPP. According to the analysis, RSPP with this capacity can reduce the annual bill of the studied mosque to a level that is equal to or lower than the annual bill in the subsidized tariff scheme. In addition to that, RSPP also provide an opportunity for carbon emission reduction. Overall, such approach can increase the effectiveness of government’s fund spending in supporting the electrification program while pursuing the target of renewable energy integration.

The need for further exploration is also supported by the large diversity of worship facilities in different areas in Indonesia that are not yet electrified. Expanding the study scope utilization of the mosques as a community center and distributed generation for renewable energy is also of further interest.

## Supplementary Information

Supplementary Information 1.

Supplementary Information 2.

## Data Availability

The datasets supporting the conclusions of this article are available in the Solcast Toolkit repository, [https://doi.org/10.25911/5c073e713e5dd, https://solcast.com].
