# Supplementary material for: Techno-economic analysis of rooftop solar power plant implementation and policy on mosques: an Indonesian case study
Source: Sci Rep. 2022 Mar 21;12:4823. doi: 10.1038/s41598-022-08968-6 (PMC8937498; doi:10.1038/s41598-022-08968-6)
Supplement: Supplementary file 1 — Supplementary Information 1. [file 41598_2022_8968_MOESM1_ESM.pdf]

Supplementary materials

Techno-economic Analysis of Rooftop Solar Power Plant Implementation and Policy on Mosque: An Indonesian Case Study

Suparwoko[1] and Fadhil A. Qamar[2]

Table S1 and S21

Table S1 PV modules data sheet

	Unit	STP375S
Price	US$	131.537
Length	m	1.756
Width	m	1.039
V oc	V	41.1
I sc	A	11.64
V mpp	V	34.7
I mpp	A	10.96
Temp. coeff P	-	-0.0036
Temp. coeff V	-	-0.00304
T noct	°C	42
Efficiency	%	20.6
Degradation (25 Y)	-	0.848
P mpp	W	375
Mass	kg	20.3
Price/power	US$/kW	350.77
Power density	W/m^2	205.5

Table S2 Inverter data sheet

No	Type	Price (US$)	Max PV Power (W)	Max OC input V (V)	MMPT V range (V)		Output Power (W)	Efficiency	US$/W
1	GROWATT MIN-750TL-X	252.6	1050	500	50	500	750	0.974	0.448
2	GROWATT MIN-1000TL-X	264.5	1400	500	50	500	1000	0.974	0.352
3	GROWATT MIN-1500TL-X	279.2	2100	500	50	500	1500	0.974	0.248
4	GROWATT MIN-2000TL-X	319.1	2600	500	50	500	2000	0.974	0.212
5	GROWATT MIN-2500TL-X	359.0	2800	550	65	550	2500	0.976	0.191
6	GROWATT MIN-3000TL-X	412.2	3500	550	65	550	3000	0.976	0.183
7	GROWATT MIN-3000TL-XE	478.7	4200	550	80	550	3000	0.982	0.212
8	GROWATT MIN-3600TL-XE	505.3	5040	550	80	550	3600	0.982	0.187
9	GROWATT MIN-4200TL-XE	545.2	5880	550	80	550	4200	0.984	0.173
